# AGMA-PESS: a deep learning-based infant pose estimator and sequence selector software for general movement assessment

**DOI:** 10.3389/fped.2024.1465632

**Published:** 2024-12-18

**Authors:** Ameur Soualmi, Olivier Alata, Christophe Ducottet, Anne Petitjean-Robert, Aurélie Plat, Hugues Patural, Antoine Giraud

**Affiliations:** ^1^ Laboratoire Hubert Curien UMR 5516, CNRS, Institut d’Optique Graduate School Université Jean Monnet Saint-Etienne, Saint-Etienne, France; ^2^ INSERM, U1059 SAINBIOSE, Université Jean Monnet, Saint-Étienne, France; ^3^ Néonatalogie, Centre Hospitalier Universitaire de Saint-Étienne, Saint-Étienne, France; ^4^ Service de Réanimation Néonatale, Centre Hospitalier Universitaire de Saint-Étienne, Saint-Étienne, France

**Keywords:** preterm infant pose estimation, automatic sequence selection, general movements, windows software, infant spontaneous movements

## Abstract

The General Movement Assessment (GMA) is a validated evaluation of brain maturation essential to shaping early individual developmental trajectories of preterm infants. To ensure a reliable GMA, preterm infants should be recorded for 30 to 60 min before manually selecting at least three sequences with general movements. This time-consuming task of manually selecting short video sequences from lengthy recordings impedes its implementation within the Neonatal Unit. Moreover, an accurate pose estimation tool for preterm infants is paramount to developing the field of GMA automation. We introduce the AGMA Pose Estimator and Sequence Selector (AGMA-PESS) software, based on the state-of-the-art deep learning infant pose estimation network, to automatically select the video sequences for GMA at preterm and writhing ages and estimate the pose of infants in 2D. Its simplicity and efficiency make AGMA-PESS a valuable tool to promote GMA use within the Neonatal Unit, both for clinical practice and research purposes.

## 1 Introduction

The general movements represent the spontaneous motor activity occurring from nine weeks of gestational age (GA) to the apparition of goal-directed movements around four months of corrected age (1). The General Movement Assessment (GMA) is a validated evaluation based on the qualitative analysis of general movements’ complexity, variability, and fluidity (1, 2). The GMA is a functional evaluation of brain maturation that reflects the integrity of extensive cortical-subcortical networks (2). The GMA performed within the Neonatal Unit is essential to shape early individual developmental trajectories (3) to initiate early developmental intervention (4).

To ensure a reliable GMA, preterm infants should be recorded for 30 to 60 min according to Prechtl’s method (1) lying in a supine position in the incubator, bed, or on the floor. If the assessment is done without acoustic signals, the assessor must be able to see the infant’s face to make sure that rigid movements are not due to crying. The room temperature should be comfortable, and in case of prolonged episodes of fussing, crying, or distraction, the recording must be stopped. Then, each video must be reviewed to manually select at least three sequences of general movements (1). The manual selection of these sequences is time-consuming and impedes the implementation of the GMA for preterm children within the Neonatal Unit. Over the past decade, multiple studies have focused on automating the assessment of general movements. This task mainly depends on accurately detecting the infant pose through the manually selected video sequences for further analysis. Openpose (5) deep learning model and its retrained version from Chambers et al. (6) were widely used in this context by numerous studies (7–11). However, Openpose was trained on datasets containing only images of adults (5), and the retrained version was generated using 9,039 infant images only, mainly collected from the internet (6), which resulted in poor performance when applied to infants’ poses in a clinical environment (12).

Recently, a new architecture was retrained on a dataset of over 88k images of infants in a clinical environment, achieving state-of-the-art results with a PCK@0.2 of 98.30% (12). Based on these results, and to address the raised problems, we developed the AGMA Pose Estimator and Sequence Selector (AGMA-PESS) software, a deep-learning-based software that automatically estimates infants’ pose and automatically selects video sequences for the GMA of preterm and term infants.

## 2 Method

**Figure 1:**
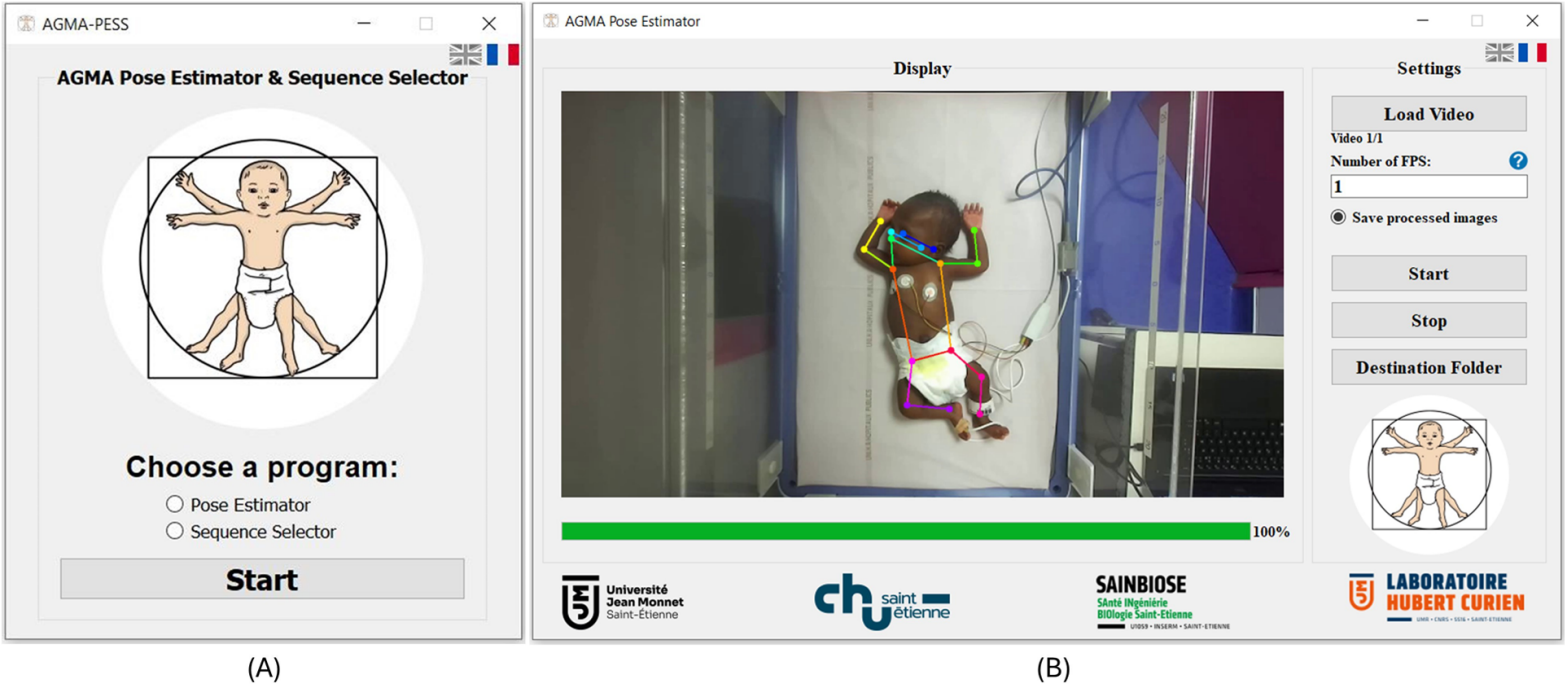
AGMA-PESS user interface. (**A**) Initialization window. (**B**) AGMA Pose Estimator window.

AGMA-PESS software was developed using Python 3.7 programming language. The graphical user interface (GUI) was created based on the Pyqt5 library. The source code can be found in this Github repository^1^ and the installation .exe file can be downloaded using this link.^2^ The AGMA-PESS is compatible with the Windows operating system (Microsoft, WA) with version ≥7. It is available in two languages (English and French). The software proposes two main functionalities to the user (see Figure 1A): a pose estimator and a sequence selector.

### 2.1 Pose estimator

Infant pose estimation is the automatic identification of key points on an infant’s body, such as joints and limbs, to understand their posture and actions (see Figure 1B). The process typically consists of using deep learning models trained on annotated datasets of infant images or videos. The neural network used in this task is the retrained version of Darkpose32 (12). This network was primarily pretrained on adult images (57 K images and 150 K person instances), then fine-tuned with a dataset containing over 88 k images of 53 preterm infants born before 33 weeks of gestational age (12). The neural network was built as a concatenation of the HRNet network (13) in addition to Darkpose (14), which was used as a plugin that improves the performance of SOTA Human pose estimation models using a new heatmap decoding process. After fine-tuning it, the network achieved a PCK@0.2 of 98.30 on 36,000 infants’ test images in the 2D pose estimation task and a mean error of 1.72 cm in the 3D pose infant estimation task. To reconstruct the infant’s 3D pose, the network estimates the 2D pose from each view of the stereoscopic images; then, a triangulation is performed to get the real-world 3D coordinates. However, since standard 2D-image acquisition systems are more popular for GMA assessment rather than stereoscopic systems, AGMA-PESS software processes 2D videos only, which does not prevent using it on each view of a stereoscopic setup if needed.

For processing efficiency considerations, AGMA-PESS offers the ability for the user to choose the number of images to process per second instead of all the video images (e.g., 30 FPS). The pose estimator window is a user-friendly interface (see Figure 1B), where one or multiple videos of any duration from the following formats (webm, .mkv, .vob, .avi, .mts, .mov, .wmv, .mp4, .mpg, .3gp, and .flv) can be loaded using only a push-button. The user will be asked to input the pose estimation frame rate as an integer value and to choose whether to save the processed images or not. The image saving format is .jpeg which offers a good balance between file size and quality. Finally, the start button will start the processing, and the user can stop it at any moment by clicking the stop button. At the end of processing, Excel files are generated automatically, where tables of the 17 keypoints coordinates and their respective confidence scores are saved and easily accessible from the “Destination folder” push button.

### 2.2 Sequence selector

The sequence selector automatically selects short video sequences where the infants exhibit the greatest amount of movement. First, the AGMA network (12) is used to estimate the infant pose (17 keypoints) $P=[p_{1},p_{2},\ldots ,p_{17}]$ through all the video (N seconds) at one frame per second rate. Next, the sum of the Euclidean distances between respective keypoints is calculated for every two consecutive poses $P_{i}$ and $P_{i+1}$, as in the following Equation 1.

(1) $$S_{i}=\sum_{k=1}^{17}\left\|P_{i+1,k}-P_{i,k}\right\|\;\text{with}\;i=1,2,\ldots ,N$$

**Figure 2:**
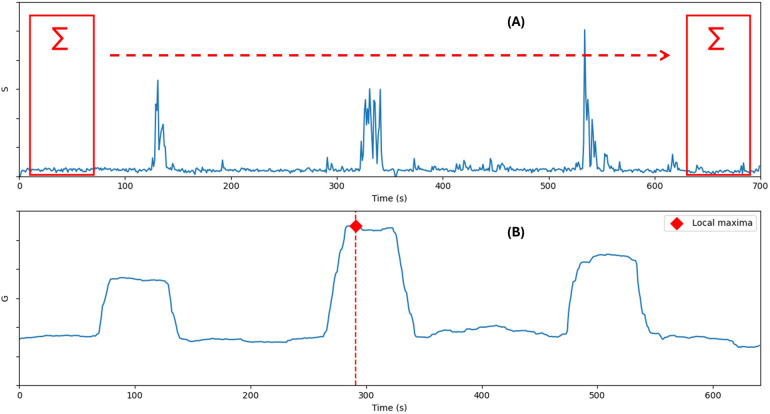
**(A)** The plot of the quantity of motion S. **(B)** The plot of the summing window function G.

$S_{i}$ is a time series that represents the quantity of motion along the video, and the task of finding the starting index x of the most important one-minute sequence of movements from a video can be described mathematically as finding the most important sub-series in $S_{i}$ (see Figure 2), which can be accomplished by a summing window function G of size 60 s as in Equation 2

(2) $$x=\underset{\;j\in 0,\ldots ,N-60}{\mathrm{argmax}}\;G(j)=\underset{\;j}{\mathrm{argmax}}\;\left(\sum_{i=j}^{\;j+60}S_{i}\right)$$

The sequence selector window has more settings than the pose estimation window. The user is asked to input the desired integer number of video sequences and their duration in minutes as a float number. When the start button is pressed, a real-time graph shows the changes in motion quantity as in Equation 1, and a progress bar shows the analysis progression. At the end of processing, the generated video sequences are accessible by clicking the “Destination folder” button.

A video tutorial is available as Supplementary Material to facilitate the use of the software.

## 3 Experiments

In order to validate the effectiveness of the proposed software, comparative experiments with manual sequence selections were conducted on a dataset of videos collected in a clinical environment.

### 3.1 Dataset

**Figure 3:**
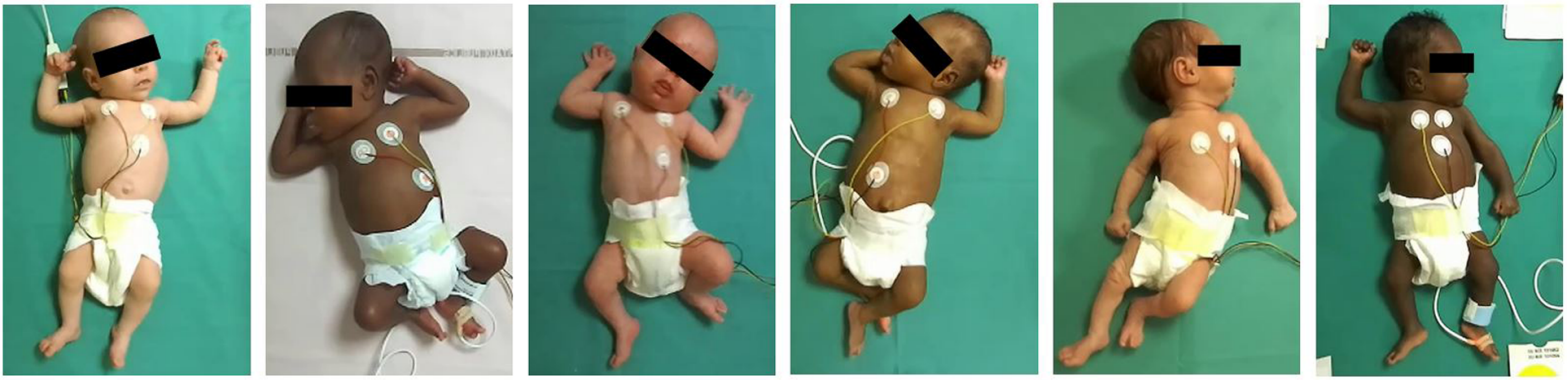
Dataset population images.

The dataset contains six video recordings of one-hour duration collected in the Neonatology department of the Centre Hospitalier Universitaire de Saint-Étienne, France. Six premature infants born before 33 weeks of gestational age (GA) were included with written parental consent. The video recording protocol was controlled as recommended in Prechtl’s method of GMA (1). Infants wearing diapers were placed in a radiant heat warmer, lying in a supine position. The room temperature was comfortable, and the recording was stopped in case of prolonged episodes of fussing, crying, or distraction. Videos were recorded for one hour using the left view of ZED2 stereoscopic camera with frames of 1,280×720 resolution at 30 FPS. The features of the recorded videos are summarized in Table 1 and some images extracted from the videos are provided in Figure 3.

**Table 1:** Dataset population features.

Infant	Birth GA (Weeks)	Term at recording (Weeks)	Video duration	Movement types	GMA
1	31	38	1 h 07 m 19 s	Writhing	Normal
2	26	34	1 h 00 m 07 s	Preterm	Abnormal
3	24	34	1 h 07 m 39 s	Preterm	Abnormal
4	31	42	1 h 01 m 11 s	Writhing	Normal
5	30	39	1 h 07 m 51 s	Writhing	Normal
6	25	35	1 h 00 m 04 s	Preterm	Normal

Abbreviations: GA, gestational age; GMA, General Movement Assessment.

### 3.2 Manual sequence selection

An expert group composed of $N_{e}=4$ experienced General Movements Trust-certified assessors (AS, APR, AP, and AG) reviewed all the video recordings. They were asked to independently select the best five video sequences by manually indicating the starting point of each sequence in seconds. The time each expert took to complete the task for each video was recorded. The results were then compared with AGMA-PESS to check the detection accuracy and validate the method.

### 3.3 Evaluation

In order to evaluate how accurately the AGMA-PESS software selects the $N_{s}=5$ GMA one-minute sequences from each of the $N_{v}=6$ videos, compared to an expert selector, the maximum dice similarity (MDS) parameter was used. Let $x_{l,i}$ with $l\in \{1,\ldots ,N_{v}\}$ and $i\in \{1,\ldots ,N_{s}\}$ be a one-minute sequence detected by the software with a time interval of $\left(x_{l,i,\mathrm{start}},x_{l,i,\mathrm{end}}\right)$. The intersection I of this sequence with another time sequence $y(y_{\mathrm{start}},y_{\mathrm{end}})$ is defined as in Equation 3.

(3) $$I(x_{l,i},y)=\max(0,\min(x_{l,i,\mathrm{end}},y_{\mathrm{end}})-\max(x_{l,i,\mathrm{start}},y_{\mathrm{start}}))$$

Since the selected intervals are different from one expert to another, for each sequence selected by the software $x_{l,i}$, the corresponding sequence $y_{c,e}$, $c\in \{1,\ldots ,N_{s}\}$, from the five sequences $y_{\;j,e}$ with $j\in \{1,\ldots ,N_{s}\}$ selected by an expert e with $e\in \{1,\ldots ,N_{e}\}$ is found as in Equation 4:

(4) $$c=\underset{\;j\in 1,\ldots ,N_{s}}{\mathrm{argmax}}\;I(x_{l,i},y_{\;j,e})$$

In the case of equality, the minimum index is chosen. Using Equation 3, the intersection of the resulting sequence $y_{c,e}$ with a time interval of $\left(y_{c,e,\mathrm{start}},y_{c,e,\mathrm{end}}\right)$ with the sequence $x_{l,i}(x_{l,i,\mathrm{start}},x_{l,i,\mathrm{end}})$ can be modeled in Equation 5:

(5) $$I(x_{l,i},y_{c,e})=\max(0,\min(x_{l,i,\mathrm{end}},y_{c,e,\mathrm{end}})-\max(x_{l,i,\mathrm{start}},y_{c,e,\mathrm{start}}))$$

Their dice similarity (DS) is defined as in Equation 6:

(6) $$\text{DS}(x_{l,i},y_{c,e})=\frac{2I(x_{l,i},y_{c,e})}{\left(x_{l,i,\mathrm{end}}-x_{l,i,\mathrm{start}})+(y_{c,e,\mathrm{end}}-y_{c,e,\mathrm{start}}\right)}$$

The DS describes the overlap between the sequences selected by the software and each expert. Therefore, checking whether a sequence detected by the software is also selected by one expert at least can be achieved by considering the MDS in Equation 7:

(7) $$\text{MDS}(x_{l,i})=\max_{e\in \left\{1,\ldots ,N_{e}\right\}}(\text{DS}(x_{l,i},y_{c,e}))$$

The values of MDS vary between 0 and 1. A null value means there is no overlap between the sequence selected by the software and the four sequences selected by the experts. On the other hand, a 1 value means that the selected sequence is also precisely selected at least by one expert. Hence, comparing the MDS to a defined threshold τ can lead to a binary classification problem, with a software-experts agreement metric defined in Equation 8:

(8) $$\text{Precision}(\tau )=\frac{\sum_{l=1}^{N_{v}}\sum_{i=1}^{N_{s}}\delta (\text{MDS}(x_{l,i})>\tau )}{N_{v}\times N_{s}}$$

where $N_{v}\times N_{s}$ is the total number of the selected sequences in all the videos.

Based on this metric, an inter-rater precision can also be calculated for each expert compared to the remaining three. This allows to verify how consistently experts make their selections, and the average inter-experts score can be used to test how well the software-experts agreement is positioned compared to the inter-experts agreement.

## 4 Results and discussion

**Figure 4:**
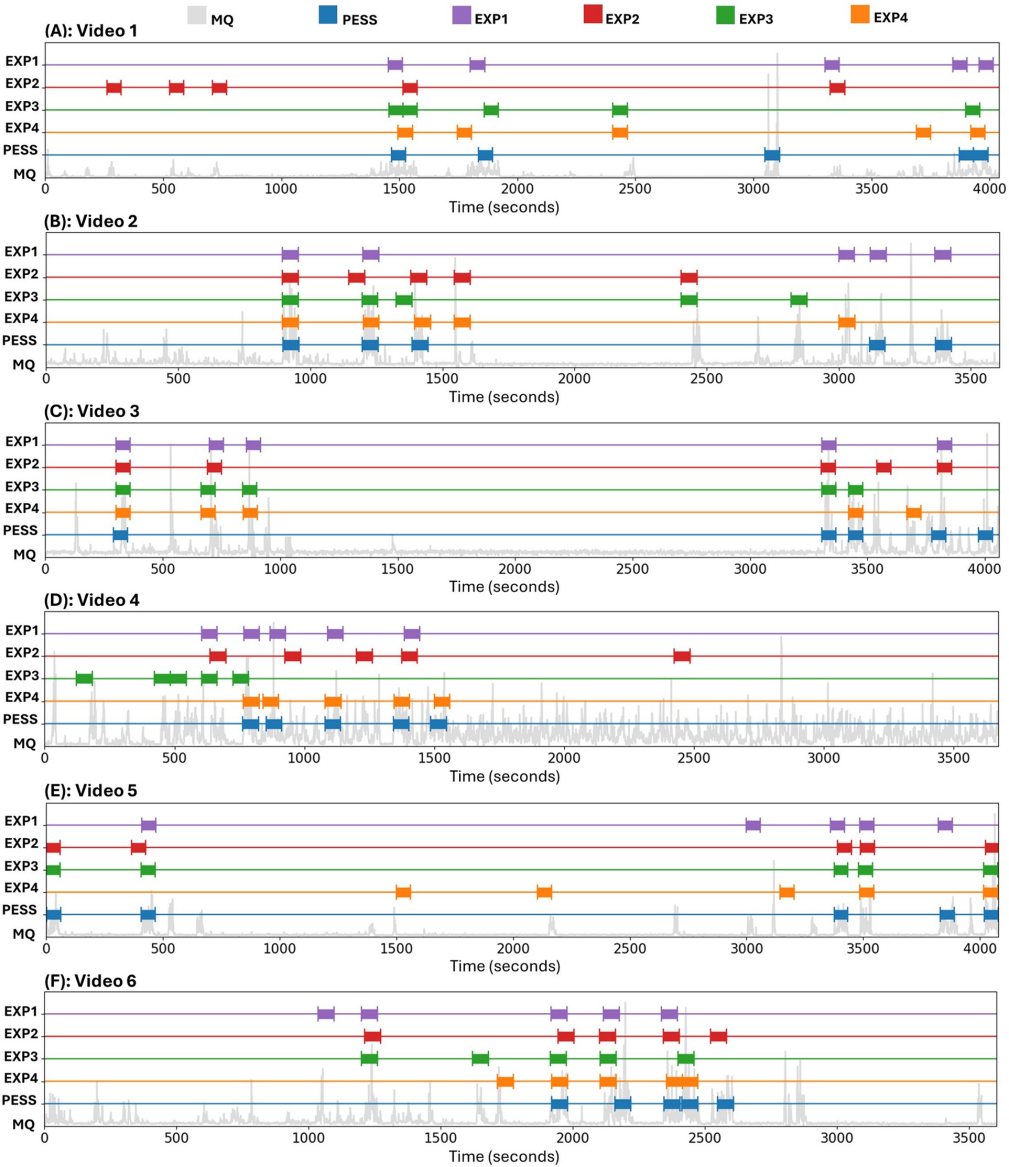
Comparison between the AGMA-PESS software and the four experts in the selection of five sequences of one-minute duration from six video recordings. Abbreviations: EXP, expert; MQ, motion quantity; PESS, AGMA-PESS software.

The comparison results between the human experts and the AGMA-PESS automatic selection of five one-minute video sequences from six videos (30 sequences in total) is illustrated in Figure 4. In 28 out of 30 cases, the sequence detected by the AGMA-PESS software intersected with a sequence selected by at least one expert, demonstrating the software’s performance relevance. Additionally, the software-expert agreement on selected sequences tended to be higher for videos where the infant was less active, such as in Video 3, which is represented by dispersed peaks in motion quantities (see Figure 4C). Conversely, in videos where the infant was very active, like in video 4, there was more variability in the selections made by the experts, creating a contrast. However, this variability is not a significant issue since all sequences selected by the experts were reliable for GMA, regardless of their location. This justifies the choice of using the maximum dice similarity parameter rather than an average of the four software-expert dice similarities, indicating that a sequence is well detected by the software if at least one expert also selects it.

**Figure 5:**
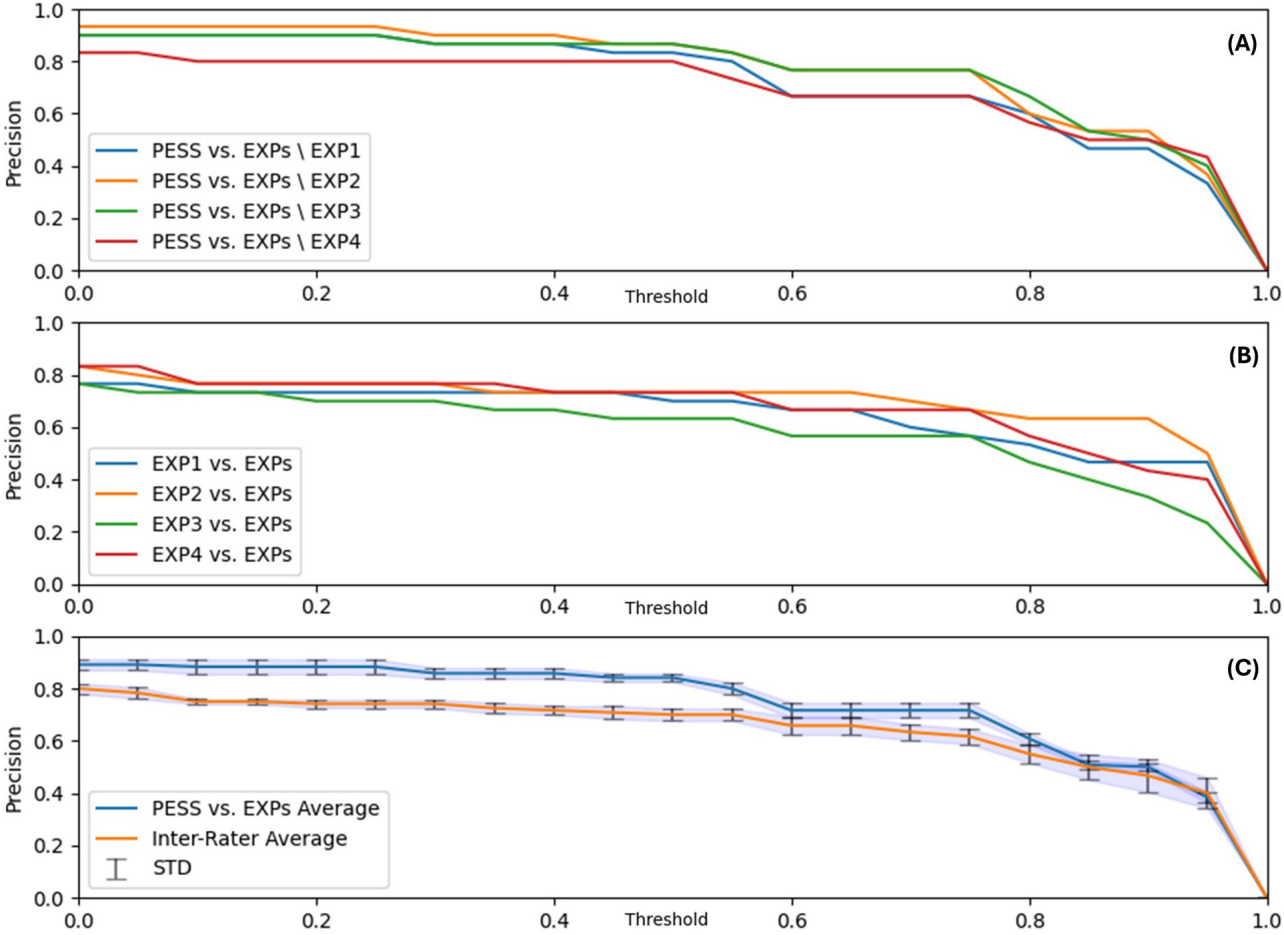
Sequence selection precision results. **(A)** Precision of the AGMA-PESS sequence selector compared to all experts except one each time. **(B)** Inter-rater agreements. **(C)** AGMA-PESS sequence selection average precision and average inter-raters agreement. Abbreviations: EXP, expert; STD, standard deviation; PESS, AGMA-PESS software.

The sequence selection precision of the AGMA-PESS software is detailed in Figure 5. In the first plot (A), and based on Equation 8, the precision of the AGMA-PESS sequence selector was calculated compared to three experts each time, resulting in four comparable graphs, which shows the relevance of the software performance. In the second plot (B), a high inter-experts agreement can be observed with varying precision for each expert. In the third plot (C), the previous graphs were averaged and compared to inspect how the software-experts agreement was positioned compared to the inter-experts agreement. With a threshold τ=0.5, which means considering a sequence as detected if it intersects with more than half with another expert-selected sequence, a precision of 86% was achieved, which is very promising since the task of manually selecting the GMA sequences can be very time-consuming (see Table 2). The average time to manually select 5 video sequences was more than 23 min, in addition to the time that will be needed to cut them for later examination. This can be automatically achieved with the AGMA-PESS software with high precision.

**Table 2:** Time spent by each expert to manually perform sequence selection.

Expert	Video 1	Video 2	Video 3	Video 4	Video 5	Video 6	Total
Expert 1	25 m 10 s	20 m 24 s	18 m 50 s	32 m 10 s	17 m 20 s	30 m 10 s	2 h 24 m 04 s
Expert 2	11 m 42 s	14 m 39 s	11 m 14 s	10 m 06 s	12 m 02 s	22 m 06 s	1 h 23 m 37 s
Expert 3	19 m 14 s	25 m 51 s	26 m 46 s	32 m 53 s	22 m 08 s	46 m 30 s	2 h 53 m 22 s
Expert 4	30 m	11 m 49 s	19 m 42 s	35 m 53 s	23 m 14 s	26 m	2 h 26 m 38 s
Average	21 m 29 s	18 m 01 s	19 m 08 s	27 m 45 s	18 m 41 s	31 m 11 s	2 h 17 m 24 s

Abbreviations: h, hours; m, minutes; s, seconds

Two sequences selected by the AGMA-PESS software had no intersection with the experts’ sequences (see Figures 4A,C). One was a valid sequence located at the end of the video. The second corresponded to a scene in which a clinician repositioned the infant, resulting in an important motion quantity. This can be interpreted as a limit of the AGMA-PESS software, which is focused on the infant’s movements and does not consider the entire scene. In addition, the software cannot differentiate the crying or fussing scenes since it is only destined for pose estimation.

For pose estimation, AGMA-PESS software achieved an average FPS (Frame per Second) speed of 2.35 (see Table 3) on a machine equipped with a GPU (NVIDIA QUADRO RTX 3000) and a CPU (Intel Core i7-10850H processor). It uses a state-of-the-art retrained network Darkpose32 (12) that has shown very accurate results for 2D pose estimation compared to existing solutions such as Openpose network. This neural network was first used in a stereoscopic framework that allowed 3D pose reconstruction (12). AGMA-PESS software analyses videos from standard 2D cameras, which are more commonly used in clinical practice. However, the AGMA-PESS software can be used on stereoscopic 3D setups by estimating 2D poses on each view, followed by manual triangulation, and selecting sequences using one view.

**Table 3:** AGMA-PESS software processing time to automatically perform sequence selection.

AGMA-PESS	Video 1	Video 2	Video 3	Video 4	Video 5	Video 6
Processing time	29 m 31 s	26 m 23 s	26 m 52 s	27 m 01 s	27 m 05 s	26 m 45 s
FPS	2.28	2.28	2.52	2.26	2.51	2.24

Abbreviations: FPS, frame per second; h, hours; m, minutes; s, seconds.

The AGMA-PESS software is user-friendly, with a video tutorial as Supplementary Material. It was tested with videos of different durations and validated to perform an automated selection of the best sequences of spontaneous motility in order to achieve the GMA of infants hospitalized in the Neonatal Unit at preterm and writhing ages from videos acquired according to Prechtl’s method of GMA (1) (infants wearing diapers in supine position). No generalization can be made about the performance of the software outside these conditions. The AGMA-PESS software was not designed to perform an automated selection of the best sequences for GMA at fidgety’s age. Nevertheless, fidgety movements should occur frequently at this age (1). The AGMA-PESS software is compatible with the Windows operating system (Microsoft, WA) with version ≥7 and is limited to non-commercial purposes.

## 5 Conclusion

The AGMA-PESS software addresses the time-consuming process of manually selecting video sequences for GMA and offers a simple GUI for estimating an infant’s pose with high accuracy. The experiments proved the software’s consistency and precision, comparable to the human level. The software’s compatibility with Windows operating systems, user-friendly interface, and availability in multiple languages enhance its accessibility and make it a valuable tool for clinicians and researchers working in neonatal care. It was extensively used in the previous study by Soualmi et al. (15) for selecting 183 one-minute video sequences and estimating the pose of the infants in these videos for automatic classification of infants’ movements. While AGMA-PESS excels in a clinical environment, its generalizability to diverse conditions remains a subject for further exploration.

## Data Availability

The raw data supporting the conclusions of this article will be made available by the authors, without undue reservation.

## Footnotes

^1^https://github.com/AmeurSoualmi/AGMA-Pose-Estimator-and-Sequence-Selector.

^2^https://drive.google.com/file/d/1y_ReeJUFWt6_V1KUSRPFYD27L-47NB5C/view?usp=sharing.
